# Prognostic Factors in Medullary Thyroid Cancer: A Real-World Study in a Referral Center

**DOI:** 10.3390/biomedicines14071431

**Published:** 2026-06-24

**Authors:** Rosa Lauretta, Giulia Puliani, Irene Terrenato, Marta Bianchini, Marilda Mormando, Marialuisa Appetecchia

**Affiliations:** 1 Oncological Endocrinology Unit, IRCCS Regina Elena National Cancer Institute, 00144 Rome, Italy; giulia.puliani@ifo.it (G.P.); marta.bianchini@ifo.it (M.B.); marilda.mormando@ifo.it (M.M.); marialuisa.appetecchia@ifo.it (M.A.); 2 Biostatistics and Bioinformatic Unit, Scientific Direction, IRCCS Regina Elena National Cancer Institute, 00144 Rome, Italy; irene.terrenato@ifo.it

**Keywords:** medullary thyroid cancer, prognostic factors, PFS, OS

## Abstract

**Background**: Several factors have been reported to influence the prognosis of medullary thyroid cancer (MTC). This study aimed to identify prognostic variables associated with progression-free survival (PFS) and overall survival (OS) in a cohort of patients treated at our institution. **Patients and Methods**: We performed a retrospective analysis of 107 consecutive patients with histologically confirmed MTC who were followed for at least 12 months. Demographic, clinical, and pathological data were retrieved from medical records. The association between baseline variables and survival outcomes was evaluated using univariate Cox proportional hazards regression models. The study was approved by the local ethics committee. **Results**: The median age at diagnosis was 56 years (range, 10–80 years), and 63% of the patients were female. Germline REarranged during Transfection (RET) mutations were identified in 10% of cases. The median follow-up duration was 100 months (range, 12–464 months). At diagnosis, disease stages were distributed as follows: stage I, 52%; stage II, 12%; stage III, 17%; and stage IV, 19%. Female patients showed significantly longer PFS compared with males (Hazard Ratio (HR) = 0.41, 95% Confidence Interval (CI) (0.21–0.82); *p* = 0.012). Factors associated with PFS by Cox regression models were post-operative serum calcitonin (CT) values after 1 and 3 months of surgery (HR = 0.08, 95% CI (0.03–0.20); *p* < 0.001; HR = 0.03, 95% CI (0.01–0.11); *p* < 0.001, respectively), Tumor, Node, and Metastasis (TNM) stage III–IV (HR = 16.86, 95% CI (5.87–48.44); *p* < 0.001), presence of lymph nodes metastasis at diagnosis (HR = 9.6, 95% CI (3.59–25.63); *p* < 0.001), multifocal disease (HR = 2.37, 95% CI (1.07–5.28); *p* = 0.034) and capsular invasion (HR = 10.72, 95% CI (4.45–25.87); *p* < 0.001). Factors associated with OS by Cox regression models were age at diagnosis (HR = 1.07, 95% CI (1.01–1.12); *p* = 0.019) and TNM Classification of Malignant Tumours stage III-IV (HR = 6.69, 95% CI (1.42–31.62); *p* = 0.016). Although lymph node metastasis and capsular invasion were not significantly associated with overall survival (*p* = 0.178 and *p* = 0.094, respectively), both variables showed a trend toward an association with OS. **Conclusions**: The study confirmed that post-operative serum CT values, male sex, lymph nodes metastasis at diagnosis, TNM stage III and IV and capsular invasion were all associated with a lower PFS. Factors associated with OS were age at diagnosis, presence of lymph nodes metastasis, TNM stage III–IV and capsular invasion.

## 1. Introduction

Medullary thyroid cancer (MTC) is a rare tumor arising from parafollicular C-cells. It accounts for 2–5% of all thyroid malignancies [1]. MTC could be sporadic or, in approximately 25% of cases, could arise in the context of multiple endocrine neoplasia type 2A and B or familial medullary thyroid cancer [2]. The current American Thyroid Association (ATA) Guidelines on MTC reported age, primary tumor stage, lymph nodes or distant metastases, and calcitonin (CT) and carcinoembryonic antigen (CEA) serum values as prognostic factors [1]. At the histopathological level, identifying key prognostic factors is crucial for guiding treatment decisions. These factors include mitotic count, Ki67 proliferative index, and tumor necrosis [3]. Stage at diagnosis is the main predictor of survival, as confirmed by the ten-year survival rate ranging from 100% to 21% moving from stage I to IV [1,4]. Calcitonin is the most reliable diagnostic and prognostic marker for MTC, even if it is not free from analytic issues or interferences [5,6]. Post-operative serum CT can predict the risk of relapse, which is low (4.9%) in the case of undetectable post-operative values [4]. CEA is usually used as a marker of dedifferentiation of MTC, also predicting a poor prognosis [7]. Apart from these clear risk factors, the role of other prognostic factors is controversial. Conflicting data are available on the role of age [8,9] and sex [10,11,12]. Overall, available prognostic factors are not always reliable, and additional markers such as procalcitonin [13] or nomogram [11] have been proposed, but not much data on a large cohort with a long follow-up are available. The aim of the study was to identify prognostic factors associated with progression-free disease (PFS) and overall survival (OS) in our cohort of patients affected by MTC.

## 2. Materials and Methods

### 2.1. Study Design and Participants

This retrospective study enrolled 107 patients affected by medullary thyroid cancer, referred to our Oncological Endocrinology Unit at the IRCCS Regina Elena National Cancer Institute, Rome. Inclusion criteria were: confirmed histological diagnosis of medullary thyroid cancer and follow-up of at least 12 months. Patients with a follow-up period of less than 13 months were excluded. All patients provided written informed consent to data collection. The study was conducted in agreement with the Declaration of Helsinki and was approved by the local review board of our Institute (RS1481/21(2464)).

### 2.2. Data Collection

For all patients, we used a standardized file for collecting the following information: age at diagnosis; sex; family history of any tumors; neoplastic comorbidities; germinal RET mutation evaluation; baseline and follow-up biochemical examination; histological examination, including tumor location and dimension, multifocality, staging, lymph node assessment, and thyroid capsular invasion; follow-up information, including the number of relapses, date(s) of progression (structural disease), and date of death; and treatment used.

### 2.3. Statistical Analysis

Variables of interest were expressed as frequencies and percentage values in the case of categorical variables and as mean ± standard deviation or median and range (minimum-maximum) in the case of continuous variables, as appropriate. The difference between the binomial proportions between groups on a dichotomous variable was assessed by the chi-square test or Fisher’s exact test, as appropriate. Normality of distribution was assessed by the Shapiro–Wilk test. Differences in continuous variables between groups were evaluated by the Student’s *t*-test in cases of normally distributed variables and by the non-parametric Mann–Whitney test (for two groups) or Kruskal–Wallis test (for several groups) if parameters were not normally distributed. Survival analyses were performed using the Kaplan–Meier method. The Cox proportional hazards model was used to conduct univariate models to evaluate the impact of the parameters of interest on survival outcomes. A *p*-value of less than 0.05 was considered significant. All statistical analyses were performed using SPSS for Windows v.30.0 (IBM, Armonk, New York, NY, USA).

## 3. Results

Patients’ characteristics are summarized in Table 1. The median age at diagnosis was 56 (10–80) years, and 40 patients (37%) were male. Fourteen percent of patients reported a familial history of MTC, and 10% of cases harbored germinal RET mutation (20% multiple endocrine neoplasia (MEN) type 2A; 80% familial medullary thyroid cancer). The RET mutations identified were V804L, C634G, C630R, and S891A.

From the data collected, fine needle aspiration biopsy (FNAB) of thyroid nodules was available for 67 cases. Suspicion of MTC was reported in 39% and suspicion of any malignancy in 16%, while a diagnosis of benign nodules was present in 24% of cases, and in 13%, the cytological result was indeterminate. In these last two cases, patients were referred to surgery mainly for calcitonin levels, both in the needle wash liquid or in serum, or for diagnosis of MTC in lymph nodes. All patients underwent total thyroidectomy associated with central neck lymph nodes dissection and, when indicated, also with lateral neck lymph dissection. The median tumor diameter was 10 mm (3–100), and the tumor arose in the left lobe of the thyroid in 53% of cases. Most tumors were unifocal (79%), and tumor capsular invasion was present in 20% of cases. We took a value lower than 10 pg/mL as the cutoff for calcitonin negativity post-surgery. Nearly half of the patients had stage I at diagnosis, while 19% had metastatic disease, especially lymph node and liver metastases (stage I: 52%; stage II: 12%; stage III: 17%; stage IV: 19%, respectively). The median duration of follow-up was 100 months (range: 12–464). At the end of follow-up, 11 patients died (8 from the disease, 3 from other causes). Of the remaining patients, 67% were free of disease and 33% developed at least one progression.

### 3.1. Factors Associated with Progression-Free Survival

**Figure 1:**
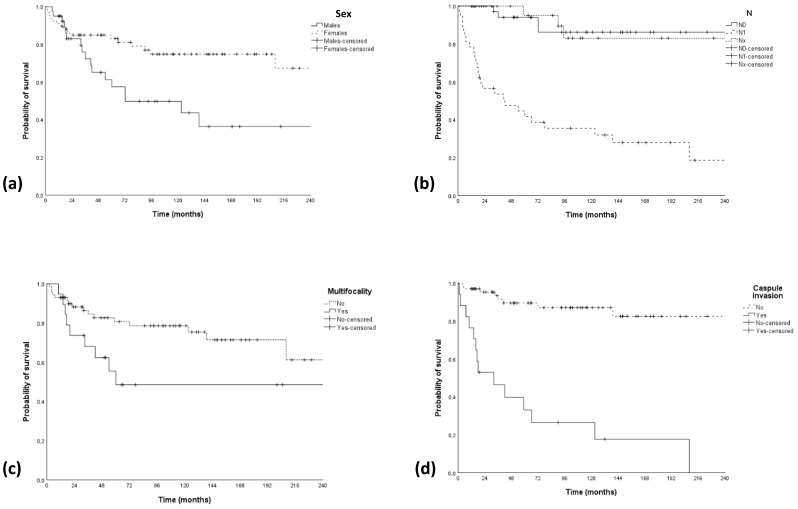
(**a**) PFS according to sex; (**b**) lymph node; (**c**) multifocality; (**d**) capsular invasion. *N*: lymph node.

Female sex was associated with longer PFS (HR = 0.41, 95% CI (0.21–0.82); *p* = 0.012) (Figure 1). Evaluating differences between males and females, we found a difference only in RET mutation prevalence, which was higher in females (*p* = 0.012). Other prognostic factors were presence of lymph node metastasis at diagnosis (HR = 9.60, 95% CI (3.59–25.63); *p* < 0.001), capsular invasion (HR = 10.72, 95% CI (4.45–25.87); *p* < 0.001), and multifocal disease (HR = 2.37, 95% CI (1.07–5.28); *p* = 0.034) (Figure 1). Other factors associated with PFS by Cox regression models were low post-operative serum CT values 1 and 3 months after surgery (HR = 0.08, 95% CI (0.03–0.20); *p* < 0.001; HR = 0.03, 95% CI (0.01–0.11); *p* < 0.001, respectively) and TNM stage III-IV (HR = 16.86, 95% CI (5.87–48.44); *p* < 0.001). Univariate analysis is reported in Table 2. A multivariate analysis was not performed due to the presence of numerous missing data that did not allow for an adequate evaluation. Unfortunately, the lack of data is related to the retrospective nature of the study.

### 3.2. Factors Associated with Overall Survival

**Figure 2:**
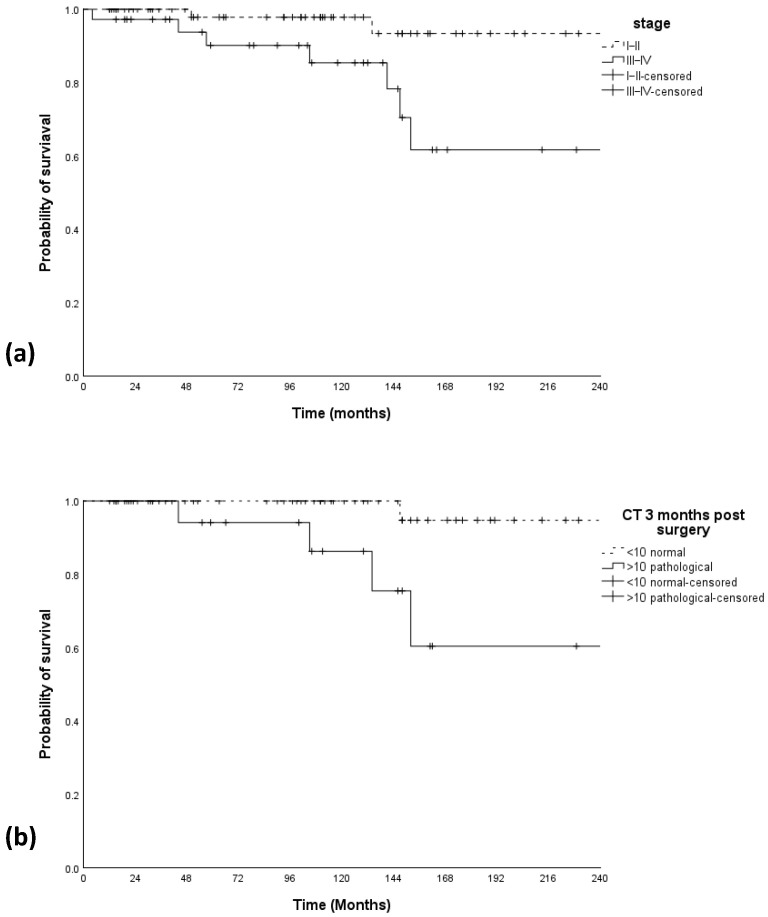
Factors associated with overall survival according to (**a**) stage, (**b**) post-operative CT values after 3 months of surgery. *CT*: calcitonin.

Factors associated with OS disease related correlated by Cox regression models were age at diagnosis (HR = 1.07, 95% CI (1.01–1.12); *p* = 0.019), TNM stage III-IV (HR = 6.69, 95% CI (1.42–31.62); *p* = 0.016) and low post-operative serum CT values after 3 months of surgery (HR = 0.09, 95% CI (0.01–0.8); *p* = 0.031) (Figure 2). The multifocality and the capsular invasion are not statistically significant (HR = 3.68, 95% CI (0.80–16.91); *p* = 0.094; HR = 1.44, 95% CI (0.28–7.48); *p* = 0.661), respectively) (Table 3).

## 4. Discussion

Our study confirmed the importance of the disease stage and lymph node status as prognostic factors in MTC for both PFS and OS, as extensively demonstrated in the literature. The involvement of lymph nodes, indicated by the number of positive lymph nodes and the presence of metastatic lymph nodes in multiple compartments, suggests the probability of having distant metastases. Additionally, the presence of lymph nodes in the mediastinal or lateral neck lymph nodes relative to the tumor seems to be an indicator of distant metastases [9,12,14,15,16,17,18,19,20,21,22,23,24,25,26,27,28]. The local extension of the disease and the presence of distant metastases (stage III–IV) have been identified in the literature as the main prognostic factors for MTC [9,10,15,16,19,20,22,26,27,28,29,30,31,32,33,34]. Conversely, the identification of sex and age as prognostic factors is still debated in the literature. Some studies reported that male patients seem to have a worse prognosis [8,9,12,15,18,29,35], while other studies have shown no significant differences between the sexes [16,36]. However, the specific molecular mechanisms responsible for this disparity remain unclear [12]. Multiple studies have demonstrated that the estrogen receptor is expressed in both healthy and pathological thyroid tissues in males and females. However, receptor presence does not correlate with lymph node involvement or vascular and capsular invasion in differentiated thyroid cancer [37,38,39,40,41]. In MTC, the increased aggressiveness of the tumor appears to be biologically determined in males [12]. In female tissues, a mosaic of predominantly X-linked genes is present due to X-chromosome inactivation. The response to cancer differs between the sexes with regard to X-chromosome genes [42]. It has been shown that the interaction of sex hormones with estrogen receptor alpha (ERα), estrogen receptor beta (ERβ), and the androgen receptor (AR) induces a change in gene expression within the tumor microenvironment [43]. In C-cell hyperplasia (100%) and medullary thyroid carcinoma (97%), ERβ expression was consistently found, while ERα was not expressed [44]. A simple comparison of ER receptor expression patterns is not sufficient to support the causal relationship between estrogens, their receptors, and the development of thyroid cancer [45]. In over half of patients with C-cell hyperplasia, the androgen receptor (AR) is present, occurring more commonly in males [44]. In light of higher serum androgen levels in men, we can assume that the genomic effect on the androgen receptor contributes, at least in part, to the male prevalence of C-cell hyperplasia and medullary thyroid carcinoma [44] and to higher serum calcitonin levels in men [46], although the diagnosis of MTC is less frequent in men, as in our population. Male gender was associated with a shorter PFS, in line with other reports described in the literature [9,18,32,35]. With regard to age, Matrone et al. considered an age cutoff of 65 years and found no significant differences between the two groups [8]. In contrast, Su et al. identified two age groups—those under 18 years and those over 55 years—in which age was recognized as a risk factor for distant metastases [9]. Advanced age is a significant prognostic factor for survival, as demonstrated in the literature [15,16,18,19,21,22,24,27,29,30,32,33,36,47]. In our study, age influenced only OS but not PFS. Comparing survival outcomes (PFS and OS) between hereditary and sporadic medullary thyroid carcinoma, we found no statistically significant difference. Although only 10% of our cases were hereditary, our data confirmed the results of other studies involving larger populations. Patients with sporadic and hereditary MTC, despite having different ages at onset, show similar disease-specific survival (DSS) and clinical course, depending on the stage at diagnosis [12,22,29,48]. The reduction in calcitonin levels after total thyroidectomy is a prognostic factor as it correlates with the risk of disease persistence and/or recurrence, the presence of distant metastases and therefore with survival [12,24,30,35,49,50,51,52]. Post-operative calcitonin assessment guides us in selecting the timing of imaging tests for early identification of persistence and/or recurrence of the disease. In our study, we found that low post-operative CT values at 1 and 3 months after surgery impacted progression-free survival. Additionally, low post-operative CT values observed 3 months after surgery were associated with overall survival. Lower pre-operative calcitonin levels showed a higher cure rate and prolonged survival. This is likely because calcitonin levels are closely related to tumor stage, which is the main predictor of survival [16,23,25,30,34,35]. Our study does not identify pre-operative CT as a risk factor for recurrence, but this is probably due to the presence of many missing values at baseline, which may have impacted the quality of the result. Capsular invasion is considered a negative prognostic factor because it reflects a more aggressive biological behavior and a greater capacity of the tumor to extend beyond its primary site; it was considered a histopathological surrogate of invasive tumor potential. It is frequently associated with a higher tumor stage and locoregional lymph node metastases. Furthermore, capsular invasion is often correlated with persistent or recurrent disease after surgery. MTCs that invade the capsule may exhibit microscopic extension beyond the apparent surgical margins, increasing the risk of residual disease even after apparently complete resection. Univariate regression analysis of 685 patients with medullary thyroid carcinoma from the SEER (Surveillance, Epidemiology, and End Results) database showed that multifocality and capsular infiltration were significantly correlated with distant metastases [9]. Multifocality in MTC, particularly when associated with RET mutations, has been linked to a less favorable disease-free survival (DFS). This relationship is largely explained by the underlying biology of hereditary MTC, in which germline activating mutations of the RET proto-oncogene drive the neoplastic transformation of parafollicular C cells throughout the thyroid gland. As a consequence, multiple independent tumor foci may develop synchronously, reflecting a diffuse field effect rather than a single localized neoplastic event. MEN type 2A and MEN type 2B frequently present with bilateral and multifocal disease. Multifocality may indicate a greater burden of neoplastic C-cell transformation and is often associated with earlier lymph node involvement, higher post-operative calcitonin levels, and an increased risk of persistent microscopic disease. In our series, the presence of a multifocal tumor predicts progression-free survival but not overall survival. Comparing the clinicopathological characteristics and survival of patients with and without lymph node metastases, it was found that in the group of patients with lateral lymph node metastases, overall survival and disease-free survival (DFS) were worse; in this group, more patients had higher calcitonin and CEA serum levels, multifocality, bilaterality, and capsular invasion [14,18,20,28]. The presence of capsular invasion is a predictive factor for both progression-free and overall survival, which was confirmed in our series. The limitation of our study is its retrospective design, which may introduce bias due to missing data and preclude multivariate analysis due to too many missing variables. A potential limitation of the overall survival analysis is the relatively low number of observed deaths, which may reduce the statistical power to detect significant associations. The study reported the real-world experience of an Italian referral center for neuroendocrine tumors, providing additional information on well-known and new prognostic factors, which are very useful in the clinical management of these patients.

## 5. Conclusions

This study highlights the prognostic value of sex, disease stage, capsular invasion, and lymph node metastasis at diagnosis, together with post-operative serum calcitonin levels during follow-up, in patients with MTC. Medullary carcinoma is a rare neuroendocrine tumor for which it is essential to plan prospective multicenter studies to improve molecular understanding of the tumor and establish appropriate management at diagnosis and effective follow-up by stratifying the risk of recurrence. In this view, the identification of prognostic factors for MTC that are easy to use in clinical practice is essential for patient management. It is essential to build registries that allow us to identify the correct therapeutic management and follow-up to advance personalized medicine.

## Figures and Tables

**Table 1:** Characteristics of patients affected by MTC.

	N	%
***Demographic information***		
**N° patients**	107	
**Gender**		
Male/Female	40/67	37/63
**Age at diagnosis**		
Median (min;max)	56 (10–80)	
**Cancer comorbidity**		
Yes/No	24/83	22/78
**Medullary familial history**		
Yes/No	15/92	14/86
**Other cancer familial history**		
Yes/No	20/87	19/81
**RET mutation**		
MUT/WT	10/91	10/88
NOT evaluated	6	6
***Biochemical results***		
**Pre-operative CT N = 75**		
Median (min;max)	130.7 (2.6–4584)	
**1-month post-operative CT N = 97**		
Median (min;max)	5.0 (0.5–1522)	
**3-month post-operative CT N = 78**		
Median (min;max)	2.2 (0.1–1534)	
**3-month post-operative CEA N = 83**		
Median (min;max)	1.8 (0.1–115.4)	
**Tumor stage (8 missing)**		
I	51	52
II	12	12
III	17	17
IV	19	19
**T (9 missing)**		
1	66	68
2	13	13
3	12	12
4	7	7
**Tumor diameter (mm)**		
Median (min;max)	10 (3–100)	
**Tumor location (18 missing)**		
Right lobe	39	44
Left lobe	47	53
Isthmus	1	1
Bilateral	2	2
**Multifocality (17 missing)**		
No	71	79
Yes, monolateral	8	9
Yes, bilateral	11	12
**N**		
N0	46	43
N1	37	35
Nx	24	22
**Tumor Capsular invasion (22 missing)**		
Yes/No	17/68	20/80
**Metastasis at diagnosis**		
Yes/No	1/106	1

*MTC*: medullary thyroid cancer; *CT*: calcitonin; *CEA*: carcinoembryonic antigen; *MUT*: mutated; *WT*: wild type; *pN0*: absence of lymph node metastasis; *pN1*: pathological evidence of lymph node(s) metastasis; *pNx*: unknown lymph node status.

**Table 2:** Cox regression models for PFS.

		Univariate	
	Comparison	HR (95%CI)	*p*-Value
Sex	F vs. M	0.41 (0.21–0.82)	0.012
Age at diagnosis	continuous	1.00 (0.98–1.03)	0.780
CT 1 month	Normal vs. pathological (>10 pg/mL)	0.08 (0.03–0.20)	<0.001
CT 3 months	Normal vs. pathological (>10 pg/mL)	0.03 (0.01–0.11)	<0.001
Germinal RET	Mutated vs. wild type	0.57 (0.17–1.92)	0.367
pN	N1 vs. N0	9.60 (3.59–25.63)	<0.001
	Nx vs. N0	0.84 (0.20–3.55)	0.814
Stage	III + IV vs. I + II	16.86 (5.87–48.44)	<0.001
Multifocal	Yes vs. No	2.37 (1.07–5.28)	0.034
Capsular invasion	Yes vs. No	10.72 (4.45–25.87)	<0.01

*F*: female; *M*: male; *CT*: calcitonin; *RET*: REarranged during Transfection; *PFS*: progression-free survival, *N0*: absence of lymph node metastasis; *N1*: pathological evidence of lymph node(s) metastasis; *HR*: hazard ratio, *CI*: confidence interval.

**Table 3:** Cox regression models for OS.

		Univariate	
	Comparison	HR (95%CI)	*p*-Value
Sex	F vs. M	0.33 (0.21–0.82)	0.090
Age at diagnosis	continuous	1.07 (1.01–1.12)	0.019
CT 1 month	Normal vs. pathological (>10 pg/mL)	0.30 (0.07–1.23)	0.094
CT 3 months	Normal vs. pathological (>10 pg/mL)	0.09 (0.01–0.80)	0.031
Germinal RET	Mutated vs. wild type	0.04 (0.17–1.92)	0.369
pN	N1 vs. N0	2.51 (0.66–9.53)	0.178
	Nx vs. N0	Not estimable **	-
stage	III + IV vs. I + II	6.69 (1.42–31.62)	0.016
Multifocal	Yes vs. No	1.44 (0.28–7.28)	0.661
Capsular invasion	Yes vs. No	3.68 (0.80–16.91)	0.094

*OS*: overall survival; *F*: female; *M*: male; *CT*: calcitonin; *RET*: REarranged during Transfection; *N0*: absence of lymph node metastasis; *N1*: pathological evidence of lymph node(s) metastasis; *HR*: hazard ratio, *CI*: confidence interval ** no deaths in the Nx group.

## Data Availability

Data analyzed in this review are included in this article. Further inquiries can be directed to the corresponding author.

## Abbreviations

The following abbreviations are used in this manuscript.

MTC	Medullary thyroid cancer
PFS	Progression-free survival
OS	Overall survival
CT	Calcitonin
CEA	Carcinoembryonic antigen
RET	REarranged during Transfection
HR	Hazard ratio
CI	Confidence interval
TNM	Tumor, Node, and Metastasis
FNAB	Fine needle aspiration biopsy
WT	Wild type
Mut	Mutated
DSS	Disease-specific survival
DFS	Disease-free survival
MEN	Multiple endocrine neoplasia
